# A new family of polymerases related to superfamily A DNA polymerases and T7-like DNA-dependent RNA polymerases

**DOI:** 10.1186/1745-6150-3-39

**Published:** 2008-10-04

**Authors:** Lakshminarayan M Iyer, Saraswathi Abhiman, L Aravind

**Affiliations:** 1 National Center for Biotechnology Information, National Library of Medicine, National Institutes of Health, Bethesda, MD 20894, USA

## Abstract

Using sequence profile methods and structural comparisons we characterize a previously unknown family of nucleic acid polymerases in a group of mobile elements from genomes of diverse bacteria, an algal plastid and certain DNA viruses, including the recently reported Sputnik virus. Using contextual information from domain architectures and gene-neighborhoods we present evidence that they are likely to possess both primase and DNA polymerase activity, comparable to the previously reported prim-pol proteins. These newly identified polymerases help in defining the minimal functional core of superfamily A DNA polymerases and related RNA polymerases. Thus, they provide a framework to understand the emergence of both DNA and RNA polymerization activity in this class of enzymes. They also provide evidence that enigmatic DNA viruses, such as Sputnik, might have emerged from mobile elements coding these polymerases.

This article was reviewed by Eugene Koonin and Mark Ragan.

## Introduction

Advances in structural biology have reinforced the conclusions from earlier protein sequence studies that the catalytic domains of all known nucleic acid polymerases belong to four basic folds [1-4]. The most prevalent of these is the RRM (RNA-recognition motif)-like fold found in the palm domains of DNA polymerases of superfamily A, B and Y, reverse transcriptases, viral RNA-dependent RNA polymerases, DNA-dependent RNA polymerases of mitochondria and certain viruses (e.g. phage T7) and nucleotide cyclases [1,3]. More recently we showed that archaeo-eukaryotic type primases (and prim-pol proteins with both primase and DNA polymerase activity) also contain a derived version of this fold, which is further related to DNA-binding domains of certain viral replication initiation proteins and the catalytic domain of rolling circle replicator tyrosine recombinases [5]. Primase activity also independently emerged in bacterial DNAG-type primases containing the TOPRIM catalytic domain with a Rossmannoid fold [6]. RNA polymerase activity dependent on DNA- or RNA-templates additionally evolved in the double-psi-beta-barrel fold, respectively represented by the primary enzymes of cellular transcription and polymerases involved in eukaryotic gene silencing (and their phage relatives) [7]. Superfamily X and bacterial PolIII-type DNA polymerases and template-independent RNA- and DNA-terminal transferases represent the fourth independent innovation of polymerase activity [4,8]. Distinct evolutionary solutions to the priming problem and multiple independent transitions to DNA-template utilizing enzymes appear to have played a key role in the origin of nucleic acid polymerases in these different folds [5].

**Figure 1:**
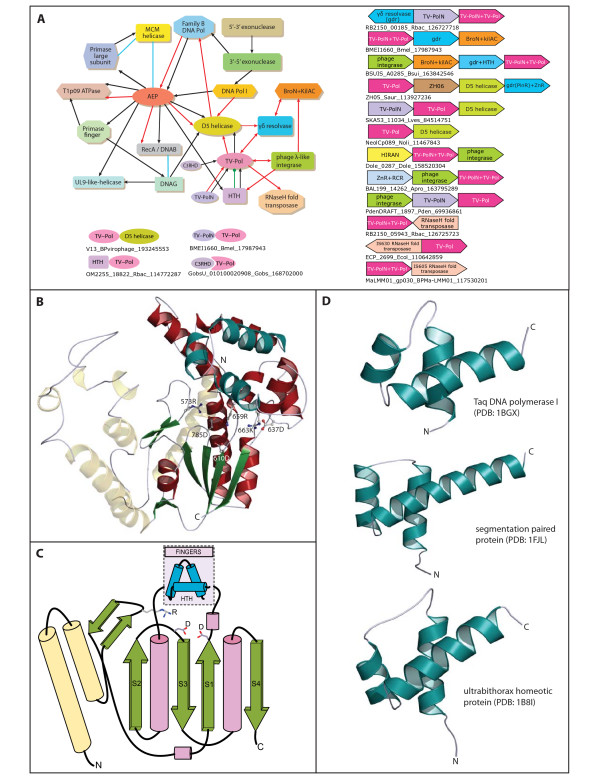
**Contextual information graph, domain architectures, gene neighborhoods, cartoon and topology diagrams**. A) Contextual information was derived from gene neighborhoods, domain architectures and functional associations (see methods used to obtain contextual information [Additional file 1]). Black arrows represent domain architectures with the arrow head pointing to the C-terminal domain. Red arrows represent gene neighborhoods, with the arrow head pointing from the 5' to 3' direction of the coding sequence. Blue edges represent physical associations and the green edge depicts domain insertion. Domain architectures of TV-Pol proteins are shown at the bottom. Gene neighborhoods (predicted operons) of TV-Pol proteins are shown to the right. Genes are represented as pointing from the 5' to the 3' end of the coding sequence. Below each gene-neighborhood cartoon is the representative TV-Pol gene name, it species abbreviation and gi. The "D5 Helicase" module includes both the core AAA+ ATPase domain and the unique D5N domain of these proteins. Likewise in the AEP module the PRICT1/2 domains are included. B) Cartoon representation of the Taq DNA polymerase (pdb: 1BGX) showing key structural units shared by TV-Pols and superfamily A polymerases. The HTH is shown in blue and the thumb domain in faded yellow. Active site and other key conserved residues are highlighted as ball and stick and labeled. C) Topology diagram of the core catalytic domain of these polymerases with key conserved features and residues. Cylinders represent helices and green arrows strands. D) Cartoon representation of various HTH domains in comparison with that seen in superfamily A DNA polymerases. Species abbreviations are in the legend to Fig. 2.

Results from comparative genomics have shown that, unlike their cellular counterparts, the universe of selfish elements comprised of viruses, plasmids and certain replicative transposons show an enormous diversity of nucleic acid polymerases. This diversity is apparent both in terms of sequence and structure of the catalytic domain, as well as domain architectures and gene-neighborhood associations of these polymerases [5,9]. This enormous sequence diversity has helped in objectively defining the core catalytic residues comprising the active sites of these enzymes and has allowed novel predictions regarding their catalytic mechanism. Concomitantly, contextual information from architectures and predicted operons has thrown considerable light on the functional partners of these polymerases. One important functional linkage which became apparent was the intimate interaction between DNA polymerases and primases of different folds with diverse DNA helicases, especially those of the ring-forming AAA+ and RecA superfamilies of P-loop NTPases [5]. Networks representing this contextual information also suggest that different families of DNA polymerases, primases, helicases and associated replication proteins frequently displace each other in different genomes or mobile elements, thereby reinforcing their functional equivalence (Fig. 1A). Hence, we utilized this phenomenon as a predictive tool to characterize novel replication enzymes and associated proteins in these elements [5].

Here, using a combination of sequence profile analysis and contextual information from genomes of viruses and other selfish elements we identify a novel family of polymerases related to superfamily A DNA polymerases and mitochondrial/phage T7-like RNA polymerases. We further present evidence that these proteins represent the minimal catalytic unit of this class of polymerases and are likely to function as both primases and polymerases, like the previously characterized prim-pol proteins.

## Results and discussion

### Identification of a novel family of nucleic acid polymerases

**Figure 2:**
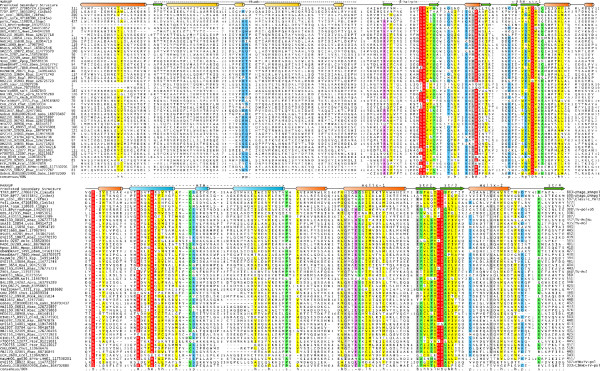
**Multiple sequence alignment of the TV-Pol domain**. Secondary structure is shown above the alignment helices shown as cylinders and strands as arrows (See additional file 1 [Additional file 1] for details on alignment and secondary structure prediction methods). The 80% consensus shown below the alignment was derived using the following classes of amino acids: the aliphatic (l: LIV, yellow shading); hydrophobic (h: ACFILMVWY, yellow shading); small (s: ACDGNPSTV, green); tiny (u: GAS, green); big (b:KMILEWRYFQ); polar (p: CDEHKNQRST, blue); charged (c: DEHKR, pink); basic (+: HKR, pink). Strongly conserved amino acids are shaded red. Sequences are denoted by their gene name followed by species abbreviation and GenBank Identifier separated by underscores. In addition to the TV-Pols some of the key superfamily A polymerases and T7-like RNA polymerases detected with significant e-values are also shown in the alignment. The species abbreviations are: Amac: *Alteromonas macleodii*; Apro: *alpha proteobacterium*; BPMa-LMM01: *Microcystis phage Ma-LMM01*; BPT7: *Enterobacteria phage T7*; BPvirophage: *Sputnik virophage*; Bmel: *Brucella melitensis*; Bsui: *Brucella suis*; Chut: *Cytophaga hutchinsonii*; Dole: *Desulfococcus oleovorans*; Ecol: *Escherichia coli*; Fbac: *Flavobacteriales bacterium*; Gbem: *Geobacter bemidjiensis*; Gobs: *Gemmata obscuriglobus*; Gpro: *gamma proteobacterium*; Gste: *Geobacillus stearothermophilus*; Lves: *Loktanella vestfoldensis*; Mgam: *marine gamma proteobacterium HTCC2143*; Mmet: *marine metagenome*; Mnod: *Methylobacterium nodulans*; Mpop: *Methylobacterium populi*; Noli: *Nephroselmis olivacea*; Oind: *Oceanibulbus indolifex*; Pden: *Paracoccus denitrificans*; Pdok: *Polaribacter dokdonensis*; Ptor: *Psychroflexus torquis*; Rbac: *Rhodobacterales bacterium*; Rbal: *Rhodopirellula baltica*; Rnub: *Roseovarius nubinhibens*; Rpal: *Rhodopseudomonas palustris*; Rsp.: *Reinekea sp*.; Rsp.: *Roseobacter sp*.; Saur: *Staphylococcus aureus*; Shae: *Staphylococcus haemolyticus*; Ssp.: *Sulfitobacter sp*.; Taqu: *Thermus aquaticus*; Tsp.: *Thauera sp*.; Vsp.: *Vibrio sp*.

D5-like proteins of the AAA+ superfamily prototyped by the poxviral D5 ATPase are the most prevalent DNA helicase encoded by genomes of medium to large DNA viruses, certain self-replicating plasmids and transposons [5]. In certain bacterial and plastid genomes (e.g. the alphaproteobacterium *Loktanella vestfoldensis*; Fig. 1A) we observed the D5-like helicase occurring in a conserved gene neighborhood with an uncharacterized gene, which encodes a globular protein (400–600 residues) with no previously identified domains. We had earlier observed that D5-like helicase domains are strongly associated with several distinct DNA replication enzymes such polymerases, primases and nucleases of the restriction enzyme fold in conserved gene neighborhoods or domain fusions in phages and prophages [5](Fig. 1A). Hence, we computationally investigated these above uncharacterized proteins linked to D5-like helicases to determine if they might have any role in DNA replication. Sequence profile searches with the PSI-BLAST program initially recovered one or more versions of related proteins from representatives of several distant bacterial lineages, namely bacteroidetes, planctomycetes, verrucomicrobia, firmicutes and proteobacteria, the cyanophage Ma-LMM01, the recently characterized Sputnik virus, the chloroplast of the chlorophyte alga *Nephroselmis *and several uncultured marine bacteria and viruses. Subsequent iterations additionally recovered superfamily A DNA polymerases of several mycobacteriophages, bacterial DNA polI and some phage T7-like DNA-dependent RNA polymerases with significant e-values (e < 10^-8^–10^-2^; see Fig. 2 legend and Additional file 1 for details). We then prepared a hidden Markov model (HMM), including all detected representatives of this uncharacterized protein family and compared it using the HHpred program to a library of HMMs prepared from all available PDB structures (see methods [Additional file 1]). This comparison retrieved the polymerase domain in HMMs derived from the Klenow fragment and Taq polymerase structures as the best hits with a p-value < 10^-5^. These searches also revealed that this family of uncharacterized proteins shared two key acidic active site residues that are essential for catalytic activity of superfamily A polymerases (Fig. 2). As these new polymerase homologs are frequently found in viral genomes or transposable elements (see below) we term these as the TV-Pol family (Transposon-Virus polymerase).

We were able to define the minimal domain of the TV-Pol family by comparing a standalone form of the protein from a rhodobacter (gi: 126727718) with the version in the V13 protein of the Sputnik virus, where it is fused to the D5-like helicase module at the C-terminus [10]. In the latter protein, this domain was previously identified as an archaeo-eukaryotic type primase (AEP) domain [10], but, as indicated by the above searches, there is no support for this relationship. We prepared a multiple alignment of the core conserved domain of the TV-Pol family using the KALIGN program, and further aligned them to superfamily A polymerases, including structurally characterized DNA PolIs and the T7 RNA polymerase (Fig. 2). The alignment showed that the minimal TV-Pol domain spanned the catalytic core of the superfamily A polymerases comprising of: 1) An N-terminal unit, the thumb module, centered on two helices in an anti-parallel coiled-coil configuration; 2) A β-hairpin supplying an absolutely conserved arginine to the active site that interacts with the template strand; 3) The core palm module formed by the RRM-like fold with two highly conserved aspartates constituting the metal-chelating active site; 4) The fingers module inserted into the RRM fold C-terminal to its first strand, with a conserved aspartate and a RxxxK motif (where x is any amino acid) (Fig. 1B, 2). Using a combination of structure similarity searches and profile-profile comparisons we were able to show that the conserved core of the fingers module of the classical superfamily A DNA polymerases, T7-like RNA polymerases and the TV-Pol family is a helix-turn-helix (HTH) domain of the tri-helical type, similar to the version in homeodomains and cI repressors (e.g. DALI Z-score for match with ultrabithorax homeodomain of 5.5; Fig 1B, 2). The highly conserved RxxxK motif of the fingers module lies in the first helix of the HTH domain and potentially interacts with the elongating daughter strand [11]. Taken together, these observations suggest that the TV-Pol domain contains all necessary features to function as a nucleic acid polymerase.

### Contextual associations implicate the TV-Pol family in transposon and virus replication

Other than in standalone forms, the TV-Pol minimal domain is combined with two other globular modules in mutually exclusive domain architectures, namely a C-terminal D5-like helicase or an N-terminal uncharacterized globular domain. Majority of D5-like helicases occurring in multidomain proteins are fused at the N-terminus to either AEP or DNAG domain primases [5] (Fig. 1A). The former domains either function exclusively as primases or as prim-pols with both primase and DNA-polymerase activities [5,9]. Searches show that C-terminal D5-like helicase modules fused with the TV-Pol domain are closest to those fused to the prim-pol family of AEPs. For example, the D5-like module fused to TV-Pol domains in an uncultured virus (gi: 144053052) and Sputnik are highly related to the D5-like domains fused to prim-pols, such as those in a selfish element from the alga *Ostreococcus lucimarinus *(gi: 145354403; e = 10^-13^) and a *Lactobacillus *phage phiadhp23 (gi: 9633023> e = 10^-11^). These associations suggest that the TV-Pol domain might displace the prim-pol module (or *vice versa*) with respect to the D5-like helicase module. Given that such non-homologous *in situ *displacements are a strong indicator of functional equivalence [5], we predict that the TV-Pol domain is likely to function, just as the prim-pols, as both a primase and polymerase. This functional linkage is also consistent with earlier noted gene-neighborhood associations observed between TV-Pols and D5-like helicases (Fig. 1A). D5-associated versions of TV-Pol are usually found in viruses or potential prophage remnants in bacterial genomes. However, in the chloroplast genome of *Nephroselmis*, the TV-Pol and D5 helicase genes occur as a linked pair in a large gene-poor, compositionally distinct, island which is absent in chloroplasts of related alga [12]. Thus, these genes might comprise a novel mobile element which has recently integrated into the *Nephroselmis *chloroplast genome.

The N-terminal uncharacterized globular domain found in a subset of the TV-Pol family is predicted to assume an all-helical fold and also occurs as a stand-alone protein encoded by a gene adjacent to the TV-Pol gene (Fig. 1A). This subset of the TV-Pol family is frequently found in conserved gene-neighborhoods (Fig. 1A) additionally containing at least one of 3 genes respectively encoding a γδ-resolvase, a phage λ-type integrase or RNAseH-fold transposase (usually either of the IS630 or IS605-type transposons). In the majority of cases these neighborhoods were embedded in genomic regions, which are hot spots for integration of several other mobile elements and restriction-modification operons and are potentially associated with negatively supercoiled DNA [Additional file 1]. This implied that these conserved gene neighborhoods coding for TV-Pol genes define novel mobile elements. These elements are reminiscent of previously uncovered elements in both prokaryotes and eukaryotes which encode AEP- or DNAG-type primase and family B DNA polymerase domains [5,13]. Consistent with this proposal we obtained potential evidence for relatively recent transposition events in *Brucella*. For example, *B. melitensis *shows two closely related, distantly located, copies (identical in protein sequence and nearly identical in DNA sequence over 4000 nucleotides) of an element with three genes encoding a TV-Pol, a γδ-resolvase and a protein with Bro-N and KilA-C domains (Fig. 1A). The extremities of this element were found to be flanked by direct repeats of about 200 nucleotides, which is comparable to the long direct repeat containing target sequences used by members of the γδ-resolvase family [14]. Furthermore, we also found a TV-Pol gene in the mobile *SCCmec *elements, which confer resistance to methicillin in *Staphylococcus*. Here too the TV-Pol gene is linked to a gene (ccrC) encoding a distinctive γδ-resolvase family protein, which has been shown to be required for the transposition of *SCCmec *elements [15,16]. Based on these observations we propose that these elements are replicative transposons, whose TV-Pol proteins catalyze priming and DNA synthesis in conjunction with the integrase or resolvase activity also coded by the element.

### Evolutionary implications of the TV-Pol family

Unlike classical DNA polI family proteins from bacteria and larger DNA viruses, TV-Pols never show fusions or conserved gene-neighborhood linkages to nuclease domains. As TV-Pols are predominantly found in mobile elements and relatively small DNA viruses, it is likely that they emerged as replicative enzymes of small ancient mobile DNA elements with little selective pressure for proof-reading activities. In their subsequent evolution TV-Pols largely remained restricted to such elements, which widely disseminated across bacterial, plastid and phage genomes. There is no evidence that in any of these cases TV-Pols displaced endogenous replication systems because these genomes are associated with the usual complement of replication enzymes including DNA polymerases. However, in the case of the Sputnik virus there are no other replicative enzymes other than the TV-Pol protein fused to the D5-like helicase module [Additional file 1]. The Sputnik virus also contains a phage integrase-type enzyme (ORF V11) and a transposase-type HTH domain, which are related to the gene-products of different TV-Pol containing elements [10] (Fig. 1A). This raises the possibility that the core precursor of the Sputnik virus was a TV-Pol containing transposase, which subsequently acquired a DNA-packaging HerA-FtsK ATPase and virion proteins from a distinct viral source. Our identification of the TV-Pol-containing element integrated in the genome of the *Microcystis *phage Ma-LMM01 provides a possible model for acquisition of viral genes by such elements.

Both the association of TV-Pols with relatively small DNA elements and presence of a minimal version of the polymerase domain in them suggest that they are close to the ancestral state of the superfamily A DNA polymerases and related T7-like RNA polymerases. An examination of the four synapomorphies which unite all these polymerases (which correspond to the four conserved features described above) suggests how the progenitor of these polymerases, which resembled the TV-Pols, emerged from the ancestral RRM-like catalytic core shared by palm units of diverse polymerases [1-3]. The thumb element was derived from a simple bi-helical anti-parallel coiled-coil, whereas an N-terminal extension in the form of a β-hairpin with a conserved arginine generated the element linking the template strand to the active site (Fig. 1B). Finally, the well known HTH DNA-binding domain appears to have been recruited as the "fingers" element by these polymerases (Fig. 1C). It is conceivable that in the earliest stages the coiled-coil unit, the HTH and the RRM-like fold of the palm functioned as independent polypeptides, which combined to give rise to a TV-Pol like polymerase domain. In favor of this proposal we observed that in a single TV-Pol from *Gemmata obscuriglobus *(gi: 168702000) the usual coiled-coil thumb module has been displaced by a distinct globular domain with conserved cysteines (Fig. 1A). This predicted Zn chelating domain is found as a standalone protein in several viral genomes [Additional file 1].

T7-like RNA polymerases have been derived from superfamily A DNA polymerases as they share certain distinct shared elements absent in the TV-Pols [17,18]. T7-like RNA polymerases, in addition to functioning as transcription enzymes in viruses, also function as primases in lagging strand synthesis in eukaryotic mitochondria [19]. Likewise, the bacterial PolI functions in filling in gaps after removing RNA primers from Okazaki fragments [18]. Mutant PolIs that catalyze RNA polymerization have also been isolated [20]. These functions can now be understood on the basis of an ancestral TV-Pol-like enzyme, which is proposed to have possessed both primase (RNA polymerase) and DNA polymerase activities. DNA PolIs appear to have expanded the ancestral DNA polymerase activity by acquiring additional domains with proof-reading and primer removing nuclease activities as they adapted to DNA synthesis in larger DNA viruses and bacterial cells. T7-like polymerases instead expanded the ancestral primase function to evolve into full-fledged RNA polymerases.

## Reviewers' comments

*Eugene V. Koonin, National Library of Medicine at the National Institutes of Health, USA *This is a very interesting, carefully conducted study that delineates a new family of palm-domain DNA/RNA polymerases. The conclusion, on the strength of contextual analysis, that these enzymes function as "primpols" appears fully justified. The hypothesis that certain DNA viruses with relatively small genomes, such as the recently discovered Sputnik, might have evolved from mobile elements containing polymerases of this new family is more on the speculative side, but plausible and of definite interest.

Mark Ragan, Institute for Molecular Bioscience, The University of Queensland, Australia

I support publication of this manuscript.

## Competing interests

The authors declare that they have no competing interests.

## Authors' contributions

LMI and LA were involved in the discovery process and writing the paper. The figures were prepared by SA. All authors read and approved the final manuscript.

## Supplementary Material

Additional file 1 Material and methods and a complete list of conserved gene neighborhoods and comprehensive alignment of the TV-Pol, TV-PolN and C3RHD domains are provided. They can be accessed from: Click here for file
