# Supplementary material for: A new family of polymerases related to superfamily A DNA polymerases and T7-like DNA-dependent RNA polymerases
Source: Biol Direct. 2008 Oct 4;3:39. doi: 10.1186/1745-6150-3-39 (PMC2579912; doi:10.1186/1745-6150-3-39)
Supplement: Additional file 1 — Material and methods and a complete list of conserved gene neighborhoods and comprehensive alignment of the TV-Pol, TV-PolN and C3RHD domains are provided. They can be accessed from: [file 1745-6150-3-39-S1.html]

A new family of DNA polymerases related to family A DNA polymerases and T7 DNA-dependent RNA polymerases.
